# The impact of maternal infection with *Mycobacterium tuberculosis* on the infant response to bacille Calmette–Guérin immunization

**DOI:** 10.1098/rstb.2014.0137

**Published:** 2015-06-19

**Authors:** Patrice A. Mawa, Gyaviira Nkurunungi, Moses Egesa, Emily L. Webb, Steven G. Smith, Robert Kizindo, Mirriam Akello, Swaib A. Lule, Moses Muwanga, Hazel M. Dockrell, Stephen Cose, Alison M. Elliott

**Affiliations:** 1 MRC/UVRI Uganda Research Unit on AIDS, PO Box 49, Entebbe, Uganda; 2 Makerere University College of Health Sciences, PO Box 7072, Kampala, Uganda; 3 London School of Hygiene and Tropical Medicine, Keppel Street, London WC1E 7HT, UK; 4 Entebbe Hospital, PO Box 29, Entebbe, Uganda

**Keywords:** maternal infection, mycobacteria, bacille Calmette–Guérin, purified protein derivative, tuberculosis, immunization

## Abstract

Bacille Calmette–Guérin (BCG) immunization provides variable protection against tuberculosis. Prenatal antigen exposure may have lifelong effects on responses to related antigens and pathogens. We therefore hypothesized that maternal latent *Mycobacterium tuberculosis* infection (LTBI) influences infant responses to BCG immunization at birth. We measured antibody (*n* = 53) and cellular (*n* = 31) responses to *M. tuberculosis* purified protein derivative (PPD) in infants of mothers with and without LTBI, in cord blood and at one and six weeks after BCG. The concentrations of PPD-specific antibodies declined between birth (median [interquartile range (IQR)]) 5600 ng ml^−1^ [3300–11 050] in cord blood) and six weeks (0.00 ng ml^−1^ [0–288]). Frequencies of PPD-specific IFN-γ-expressing CD4^+^T cells increased at one week and declined between one and six weeks (*p* = 0.031). Frequencies of IL-2- and TNF-α-expressing PPD-specific CD4^+^T cells increased between one and six weeks (*p* = 0.019, *p* = 0.009, respectively). At one week, the frequency of PPD-specific CD4^+^T cells expressing any of the three cytokines, combined, was lower among infants of mothers with LTBI, in crude analyses (*p* = 0.002) and after adjusting for confounders (mean difference, 95% CI −0.041% (−0.082, −0.001)). In conclusion, maternal LTBI was associated with lower infant anti-mycobacterial T-cell responses immediately following BCG immunization. These findings are being explored further in a larger study.

## 1. Introduction

Bacille Calmette–Guérin (BCG) is the only vaccine against tuberculosis (TB) currently available, but its protective efficacy varies between populations. Meta-analyses of trials of BCG immunization have shown that latitude is an important factor in the protection achieved in adolescents and adults [1–3]. This is an observation of great public health concern, since TB is a major cause of morbidity and mortality in developing tropical countries, where its endemicity is very high [4].

One hypothesis that has been advanced to explain the variability in BCG efficacy, and its relationship to latitude, is that sensitization to non-tuberculous mycobacteria (NTM), which is more common in lower latitudes [5], modifies the protection induced by BCG [6]. Exposure to NTM might block the induction of a protective effect by BCG, or might provide equivalent protection to BCG, obscuring the benefit provided by BCG. However, the hypothesis that exposure to NTM confers protection does not seem consistent with the high incidence of TB in tropical countries. It is recommended for BCG to be given at birth in low income settings [7]. In Uganda, hospital-born infants are immunized within hours of birth, before discharge [8]. In studies where BCG is administered early in life, BCG induces strikingly different profiles of immune response in Africa compared with the UK [9]. For infants immunized some months after birth, prior sensitization has been attributed to early exposure to environmental mycobacteria [10]. This is likely to contribute, but we postulate that intrauterine exposures may result in a more significant modification in the profile of response (which may indeed be reflected in the response to subsequent exposure to environmental mycobacteria, to BCG, or to TB).

Latent *Mycobacterium tuberculosis* infection (LTBI) is thought to involve a dynamic relationship between mycobacteria and the immune system. Individuals with LTBI may have circulating antigen and higher concentrations of TB-specific antibodies than those without infection. Mycobacterial antigens have been found to cross the placenta in murine models [11]. Thus, maternal LTBI might lead to exposure to mycobacterial antigens *in utero* and the development of a modified profile of sensitization [12], or the induction of tolerance [13,14] in the fetus. Alternatively, the passive transfer of maternal anti-mycobacterial antibodies, by providing passive immunity, might interfere with the ability of the BCG vaccine to elicit protective cellular responses. Maternal LTBI could also influence the maternal and placental immunological milieu, and hence the fetal and neonatal response on exposure to immunization [15].

We therefore propose the hypothesis that maternal LTBI influences the neonatal response to BCG (and to *M. tuberculosis*), rendering the response to BCG less effective and susceptibility to TB greater.

In Uganda, where the annual incidence of tuberculous infection is estimated at 3%, up to 60% of young women of childbearing age are likely to be infected. We present results of a pilot study to investigate the progression of immune responses to mycobacterial antigen, and the relationship between maternal infection with *M. tuberculosis* and infant immune responses, following BCG immunization at birth.

## 2. Material and methods

### (a) Study design and setting

We investigated healthy infants of mothers with and without LTBI. Women residing within the study area (Entebbe Municipality and Katabi sub-county, Wakiso district, Uganda) and delivering in Entebbe General Hospital were eligible for inclusion. Pregnant women were given prior information about the study during antenatal visits. On admission in early labour they were approached for consent if they were willing to participate in the study, had a normal singleton pregnancy and were HIV negative (based on antenatal records). Following consent, cord blood was obtained at delivery. After delivery, a brief questionnaire was completed and BCG immunization was given to the neonates before discharge from hospital. A single batch of the BCG vaccine, BCG-Russia (BCG-1 Moscow strain, Serum Institute of India, India) was used. BCG was administered intradermally for all infants within 48 h of birth.

Neonates were excluded if cord blood was not obtained, the delivery was complicated, birth weight was below 2500 g, or if the neonate presented with significant congenital abnormalities or was clinically unwell, as judged by the midwife.

Mothers were asked to return to the clinic one week after delivery. At this time, a maternal blood sample was obtained for investigation of LTBI by T-SPOT.TB assay (Oxford Immunotec, Abingdon, UK) and a tuberculin skin test (TST; 2 tuberculin units, Statens Serum Institut, Copenhagen, Denmark) was performed. This was read between 48 and 72 h later and was defined as positive if greater than or equal to 10 mm in diameter. Mothers were regarded as LTBI-positive if both T-SPOT.TB and TST were positive, and LTBI-negative if both were negative. A positive response to ESAT-6 and CFP-10 in the T-SPOT.TB was considered likely to represent *M. tuberculosis* infection in this setting, although a small number of other mycobacterial species do express these antigens [16–19].

A repeat HIV test was also performed using the standard rapid test algorithm (usually Determine (Inverness Medical, Tokyo, Japan) confirmed by HIV 1/2 STAT-PAK Dipstick test (Chembio Diagnostic Systems, Medford, NY, USA) with Uni-Gold HIV test (Trinity Biotech plc, Bray, Ireland) as a tie-breaker). Mothers with LTBI were investigated for active TB based on symptoms, sputum examination (if available) and chest X-ray. Mother–baby pairs were excluded if T-SPOT.TB and TST results were discordant or if the mother was found to be HIV-positive.

Peripheral venous blood was obtained from each infant at one and six weeks after BCG immunization. The number of infants included in this pilot study was chosen to be feasible within the time frame and resources available, and analyses were restricted to infants who had relevant results at all time points.

### (b) ELISA for anti-PPD and anti-tetanus toxoid total IgG antibodies

Total plasma immunoglobulin(Ig)G specific for PPD and tetanus toxoid (TT) was assayed using an ‘in-house’ indirect enzyme-linked immunosorbent assay (ELISA). Briefly, flat-bottomed 96-well Microlon plates (Greiner Bio-one, Germany) were coated with purified IgG standard (GenScript, NJ, USA) in bicarbonate coating buffer at maximum concentration of 0.625 µg ml^−1^ and minimum concentration of 0.01 µg ml^−1^, and PPD (10 µg ml^−1^, RT 50, Statens Serum Institut, Copenhagen, Denmark) or TT (12.12 Lf/ml, T155-1, Statens Serum Institut, Copenhagen, Denmark). Each PPD and TT well had a control comprising 0.1% Marvel milk powder (Premier International Foods, UK) in coating buffer. After overnight incubation, the plates were blocked with 150 µl well^−1^ of 1% milk powder/PBS for 1 h at room temperature. Samples diluted 1 in 100 in 0.1% milk powder/PBS were added to the plates and left overnight at 4°C. Polyclonal anti-human IgG horseradish peroxidase (Poly HRP, 0.5 µg ml^−1^, Dako, Denmark) was added at 50 µl well^−1^ and plates incubated for 1 h at room temperature. A total of 100 µl well^−1^ of o-phenylenediamine (OPD, Sigma-Aldrich, MO, USA) substrate mixture (3 mg OPD, 0.1 M citric acid, 0.2 M Na_2_HPO_4_, 3 µl 30% hydrogen peroxide in distilled water) was added for 15 min at room temperature (in the dark). The reaction was stopped with 25 µl well^−1^ 2 M sulfuric acid and the plates were read at test wavelength 490 nm and reference wavelength 630 nm using an MRX1.1 plate reader and Gen5 1.07 software (BioTek Instruments, Inc., VT, USA). The sensitivity of the test was determined as the lowest standard concentration above which antibody concentrations were detectable (0.01 µg ml^−1^).

### (c) Separation of mononuclear cells

Mononuclear cells were isolated from cord blood, and from infant blood obtained at one and six weeks after birth, by standard Ficoll-Paque (Sigma-Aldrich) density gradient centrifugation, and cryopreserved in 50% fetal calf serum (FCS) (Sigma-Aldrich), 40% RPMI 1640 medium (Life Technologies Corporation, NY, USA) containing 2 mM l-glutamine, 100 U ml^−1^ penicillin G, 100 µg ml^−1^ streptomycin sulfate and HEPES and 10% dimethylsulfoxide (DMSO) (Sigma-Aldrich) according to standard protocols.

### (d) Intracellular cytokine staining assay

The cells were thawed and rested for 24 h at 37°C in 5% CO_2_, viability and numbers were checked using Cellometer Vision Cell Profiler (Nexcelom Bioscience, LLC-Lawrence, MA, USA), and cells were stimulated with *M. tuberculosis*-purified protein derivative (PPD, 20 µg ml^−1^, RT 50, Statens Serum Institut, Copenhagen, Denmark). Medium alone was used as a negative control, and staphylococcal enterotoxin B (SEB, 200 ng ml^−1^, Sigma-Aldrich) served as a positive control. The co-stimulatory antibodies anti-CD28 and anti-CD49d (1 µg ml^−1^, BD Biosciences, San Jose, CA, USA) were included in all conditions. The cells were incubated at 37°C in 5% CO_2_ for a total of 24 h. Brefeldin A (10 µg ml^−1^, Sigma-Aldrich) was added to SEB wells after 2 h, and to PPD and negative control wells after 20 h. The cells were then stained with aqua viability dye (Life Technologies, OR, USA), and antibodies directed against the following human molecules: CD3 (QDot655, S4.1) and CD4 (QDot605, S3.50), both obtained from Life Technologies, OR, USA; CD8 (BV570, RPA-T8, BioLegend, San Diego, CA, USA); and IFN-γ (APC, B27), TNF-α (PE-Cy7, Mab11) and IL-2 (FITC, 5344.111), all obtained from BD Biosciences, NJ, USA. Stained cells were acquired on an LSRII flow cytometer (BD Biosciences, NJ, USA). Flow cytometry was not performed blinded to the maternal LTBI status, but the infants' samples were tested in a randomized sequence to limit the possibility of bias in the results due to day-to-day variation in the assay.

### (e) Statistical analysis

Antibody concentrations were summarized using medians and interquartile ranges (IQR) and compared between different time points using paired *t*-test. Antibody levels showed a skewed distribution with large numbers of undetectable results, therefore results were transformed to log_10_ (antibody concentration + 1) for graphical presentation. Flow cytometry data were analysed using FlowJo v. 9.5.2 (Tree Star Inc., Ashland, OR, USA). Results were expressed as the frequency of positive events above the negative control. Prism v6.0e (GraphPad software, Inc., La Jolla, CA, USA) was used for crude analyses and data presentation. Characteristics of mothers with and without LTBI were compared using the Mann–Whitney *U*-test. Responses at different time points were compared using the Wilcoxon signed-rank test. Differences in responses between LTBI exposed and unexposed infants were analysed using the Mann–Whitney test. Stata v. 13.0 (College Station, TX, USA) was used for multivariable linear regression to adjust for potential confounders (maternal age, gravidity status and infant gender), with 95% confidence intervals (CI) estimated by bootstrapping. Only three mothers had helminth infections and therefore this was not included as potential confounder. Results from regression analyses were presented as crude and adjusted mean difference (95% CI). *p*-Values of less than 0.05 were considered statistically significant.

## 3. Results

### (a) Participant characteristics

**Figure 1.**
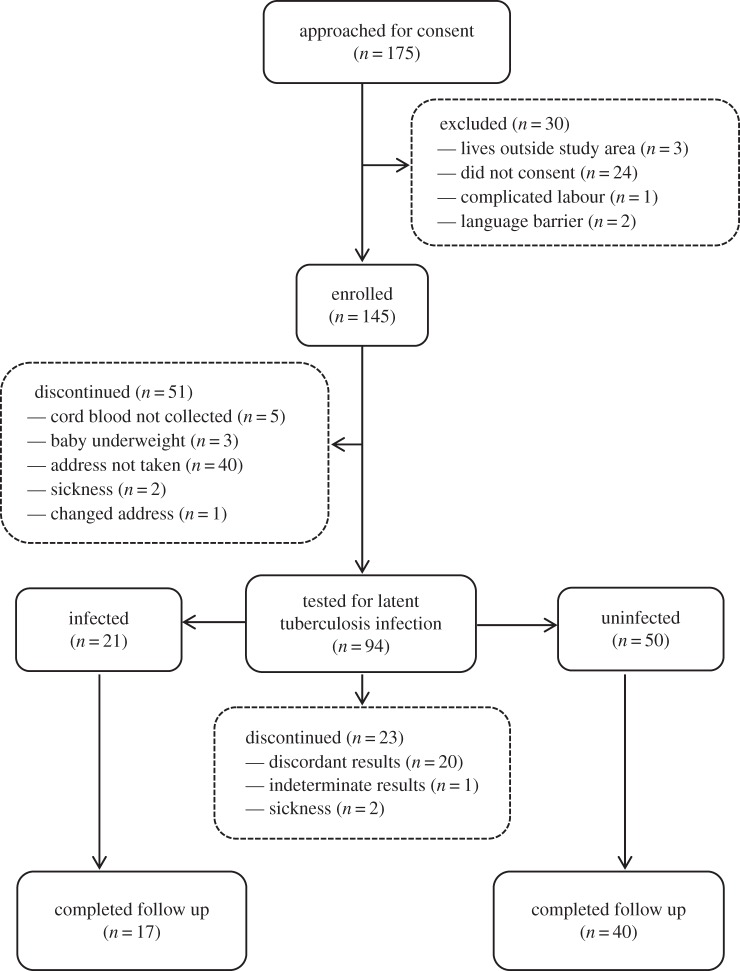
Flow of participants through the study.

Between February and May 2012, 175 women were approached to participate and 145 were enrolled in the study (figure 1). Fifty-one enrolled mothers were excluded, in most cases because contact details were not taken and they could not be traced when they defaulted from further follow-up. The remaining 94 mothers were tested for LTBI. Of these 23 were excluded, in most cases for discordant LTBI results. Twenty-one mothers were identified as LTBI-positive and 50 as LTBI-negative. None of the mothers was found to be infected with HIV on repeat testing.

Mothers with LTBI, compared to those without, were older (mean age 27.5 versus 23 years, *p* = 0.002), less likely to be primigravida (32% versus 48%, *p* < 0.001) and less likely to have a BCG scar (47% versus 70%, *p* < 0.001), and their children were more likely to be male (53% versus 35%, *p* < 0.001).

### (b) Longitudinal changes in IgG concentration

**Figure 2.**
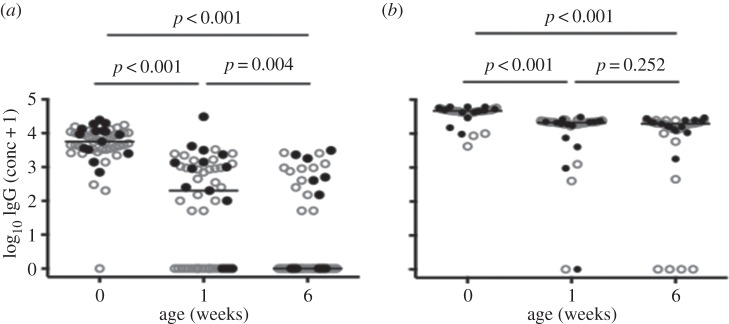
Longitudinal changes in log10 (concentration+1) PPD- (*a*) and TT-specific (*b*) IgG concentrations. Cord blood was sampled and infant plasma samples were obtained at one and six weeks; IgG concentrations were measured by ELISA. Each symbol represents an individual. Closed circles represent infants of mothers with LTBI, whereas open circles represent infants of mothers without LTBI. For each plot, the horizontal line represents the median; *n* = 53 for all three time points.

Plasma samples obtained from cord blood and infant samples collected at one and six weeks after neonatal BCG immunization were analysed for IgG specific for PPD and TT using ELISA. The main outcome of interest was the IgG response to PPD. TT-specific antibodies were assayed for comparison. Fifty-three samples were assayed at each time point, 15 from infants of mothers with LTBI and 38 from infants of mothers without LTBI. The distribution of concentrations of IgG specific for PPD and TT in the three sample types is illustrated in figure 2. Compared to the concentration in cord blood (median [IQR]: 5600 ng ml^−1^ [3300–11 050]), PPD-specific IgG concentrations decreased at one week after birth (175 ng ml^−1^ [0–1100], *p* < 0.001) and again at six weeks (0.00 ng ml^−1^ [0.00–288], *p* = 0.004; figure 2*a*).

By contrast, the concentration of TT-specific IgG was high in cord blood (46 750 ng ml^−1^ [42 000–49 950], dropped less dramatically by one week after birth (21 125 ng ml^−1^ [18 988–22 650] *p* < 0.001) and showed little change between one and six weeks (19 550 ng ml^−1^ [13 750–22 038] *p* = 0.252; figure 2*b*).

### (c) Longitudinal changes in frequencies of PPD-specific CD4^+^ and CD8^+^ T cells

**Figure 3.**
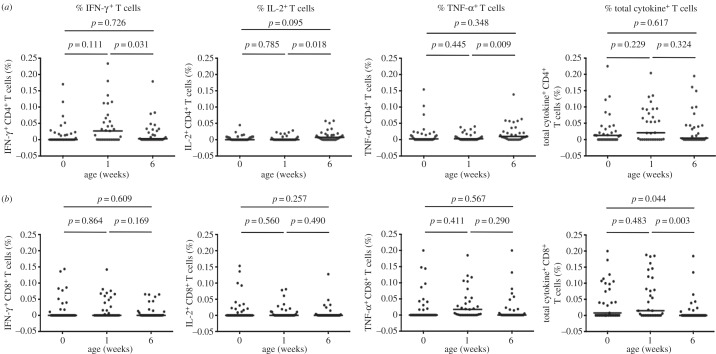
Longitudinal changes in frequencies of PPD-specific cytokine-expressing T cells during the first six weeks of life measured by intracellular cytokine staining and flow cytometry. Frequencies of PPD-specific IFN-γ^+^, IL-2^+^, TNF-α^+^ or all three cytokines combined (total cytokine^+^) CD4^+^ (*a*) and CD8^+^ (*b*) T cells. Each symbol represents an individual, and for each plot the horizontal line represents the median. Statistical analysis was performed using Wilcoxon signed-rank test; *n* = 31 for all three time points.

The frequency of CD4^+^ T cells expressing a PPD-specific IFN-γ response increased at one week and decreased at six weeks after birth, compared to responses in cord blood. The difference between frequencies at one and six weeks was statistically significant (*p* = 0.031). By contrast, the frequencies of cells expressing IL-2 and TNF-α were higher at six weeks compared to one week (*p* = 0.018 and *p* = 0.009, respectively; figure 3*a*).

The frequency of PPD-specific CD8^+^ T cells making any of the three cytokines assessed decreased at six weeks compared with one week (*p* = 0.003), and six weeks compared with cord blood (*p* = 0.044; figure 3*b*).

There was no correlation between PPD IgG concentrations and frequencies of cytokine-expressing cells in cord blood, or at one and six weeks (data not shown).

### (d) Impact of maternal infection with *Mycobacterium tuberculosis* on PPD-specific immune responses in infancy

**Figure 4.**
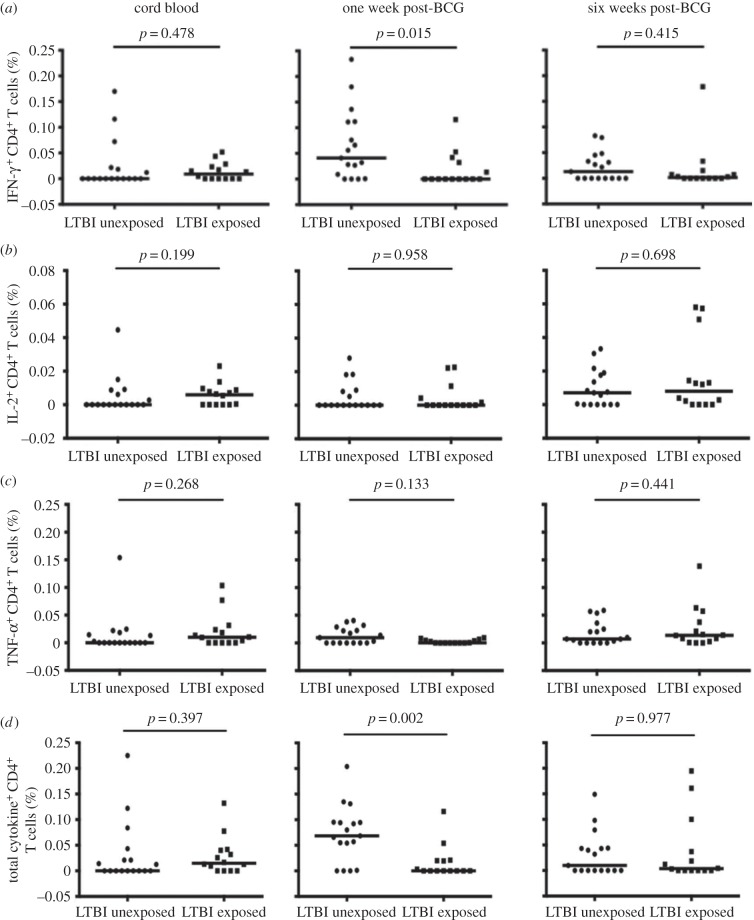
The effect of maternal LTBI on frequencies of PPD-specific CD4^+^ T cells. Frequencies for PPD-specific IFN-γ^+^ (*a*), IL-2^+^ (*b*), TNF-α^+^ (*c*) and for all three cytokines combined (total cytokine^+^ (*d*)) cells in cord blood and infant samples obtained at one and six weeks, comparing infants unexposed and exposed to maternal latent *M. tuberculosis*. Each symbol represents an individual, and for each plot the horizontal line represents the median. Statistical analysis was performed using Mann–Whitney test; *n* = 17 for infants of mothers without LTBI and 14 for infants of mothers with LTBI.

Cord blood samples obtained from infants of mothers with LTBI, compared to those without LTBI, showed a weak trend towards higher T-cell responses (*p* = 0.48 for IFN-γ (figure 4*a*), *p* = 0.20 for IL-2 (figure 4*b*), *p* = 0.27 for TNF-α (figure 4*c*), *p* = 0.40 for all cytokines, combined (figure 4*d*)). There was a similar, weak trend in cord blood PPD-specific IgG concentration (data not shown).

At one week after BCG immunization, in univariable analyses, maternal LTBI was associated with lower frequencies of CD4^+^ T cells expressing PPD-specific IFN-γ (*p* = 0.015, figure 4*a*) and of CD4^+^ T cells expressing any of the three cytokines assessed, combined (*p* = 0.002, figure 4*d*).

The mean number of cells acquired was 38 000, but 12 of the 93 samples analysed had less than 5000 events acquired. These samples came from seven infants. When affected infants were excluded from the analysis, the association between maternal LTBI and lower frequencies of CD4^+^ T cells expressing PPD-specific IFN-γ at age one week was weakened (*p* = 0.068), but that of TNF-α was strengthened (*p* = 0.045) (data not shown). Overall, the evidence for an association between maternal LTBI and infant T helper 1 (Th1) responses was therefore maintained.

In multivariable analyses incorporating all samples, after adjusting for maternal age, maternal gravidity and infant gender, the association between maternal LTBI and frequencies of IFN-γ-expressing cells was weaker than in the crude analysis (mean difference (95% confidence interval (CI)) −0.026% (−0.071, 0.019), but the association with a reduced frequency of TNF-α-expressing CD4^+^ T cells was stronger (−0.012% (−0.021, −0.003)), and the association between maternal LTBI and reduced frequencies of PPD-specific CD4^+^ T cells expressing any of the three cytokines, combined, remained strong (adjusted mean difference, 95% CI −0.041% (−0.082, −0.001)).

For CD8^+^ T cells, there was a similar, weak, association between maternal LTBI and low frequencies of PPD-specific T cells at one week (adjusted mean difference, 95% CI −0.019 (−0.042, 0.004)). There were no other differences in CD8^+^ T-cell responses between mothers with and without LTBI.

## 4. Discussion

In this study, the course of immune responses to mycobacterial antigen was studied in cord blood and in infants at one and six weeks after neonatal BCG immunization, and the effect of maternal infection with *M. tuberculosis* on the infant immune response following neonatal BCG immunization was investigated. The immune response was measured by PPD-specific plasma immunoglobulin-G concentration and frequencies of PPD-specific T cells. Two key observations emerged. First, mycobacteria-specific antibody concentrations dropped rapidly in the first six weeks of life. Second, maternal infection with *M. tuberculosis* was associated with lower infant T-cell responses one week after BCG immunization.

IgG is transferred from the mother across the placenta to the fetus by use of the neonatal Fc receptor (FcRn) [20]. Levels of passively transferred antibody to some organisms can remain high for months [21–23]. We predicted that there would be differences in concentrations of passively transferred PPD-specific antibody between infants of mothers with and without LTBI and that these differences would be sustained to at least six weeks of age. Added to this, there is evidence that BCG induces an antibody response [24] so infant antibody levels might be supplemented by those derived from the infant's own cells, post-BCG. It was therefore surprising to find that infant PPD-specific antibody concentrations dropped so markedly at one week after BCG immunization, with a further drop at six weeks such that the median response was then effectively zero. By contrast, there was only a gradual decay in maternally derived TT-specific antibodies. We speculate that the PPD-specific antibodies were lost from circulation by binding to the BCG vaccine, or to antigens released by the bacteria, and that this may affect the efficacy of the BCG vaccine. It will be important to further understand this phenomenon and to confirm its influence on BCG immunization during infancy. A randomized comparison of delayed versus immediate BCG immunization would present an opportunity to determine whether neonatal BCG immunization causes the immediate decline of passively transferred antibody and to explore these effects.

The median PPD-specific antibody concentrations in the infants of mothers with LTBI showed a tendency to be higher than for infants of mothers without LTBI. This was consistent with our hypothesis, although not statistically significant, and a larger study is needed to explore this further. If found to be significant, this would provide one possible mechanism for a difference in response to BCG immunization between infants of mothers with and without LTBI.

We report mycobacteria-specific T-cell responses following BCG immunization that are of comparable, but often lower, magnitude than those reported by Soares *et al*. [25]. In our study, the frequency of PPD-specific IFN-γ-expressing CD4^+^ T cells was highest one week after BCG immunization and decreased at six weeks, while the frequencies of PPD-specific IL-2 and TNF-α-expressing CD4^+^ T cells increased at six weeks. Soares *et al.* [25] have previously reported a peak in BCG-specific T-cell responses for each of these cytokines at 6–10 weeks after immunization. Our follow-up was too short to allow detection of a later peak. Our study differed from that of Soares *et al*. in other important respects. For example, our infants received BCG-Russia strain (Serum Institute of India, India), while Soares and colleagues provided Danish strain 1331 (Statens Serum Institut). We, and others, have shown that different BCG strains induce different responses and, in particular, that BCG-Russia induces lower initial responses than BCG Danish [26,27]. Also, we used frozen mononuclear cells (which may have resulted in lower responses compared to Soares *et al*., who used fresh whole blood [28]), stimulated cells for 24 h with PPD rather than for 12 h with BCG, and added brefeldin A at 20 h, compared with 7 h for Soares *et al*.

PPD-specific T-cell responses in cord blood tended to be higher in infants of mothers with LTBI than those born of mothers without LTBI. Although these differences were not statistically significant, they were consistent with previous studies demonstrating *in utero* sensitization to PPD among infants from mothers residing in a TB-endemic setting [12].

We found that maternal infection with *M. tuberculosis* was associated with lower infant PPD-specific CD4^+^ T-cell responses one week after neonatal BCG immunization, especially when the results for all three measured Th1 cytokines were combined. This is an observation of potential public health significance since policy in most tropical countries is for the BCG vaccine to be given at birth, and LTBI is common in these settings. The association was only seen at this early time point, and the implications of this for longer term, protective immunity are not clear. By contrast, in a recent study, Jones *et al.* [29] reported no difference in BCG-specific responses between infants exposed or unexposed to maternal *M. tuberculosis*. Again, there were important differences between our study and that of Jones and colleagues. These include the definition of maternal LTBI (both TST and T-SPOT.TB positive in our study, versus the less stringent QuantiFERON-TB Gold test positive, only, in Jones's study: the broader definition by Jones *et al.* may have contributed to smaller differences between their LTBI categories). There were differences in BCG strains used (again, Jones *et al.* used Danish strain 1331, versus BCG-Russia). Again, the schedules of the studies differed: Jones *et al.* immunized their infants at six weeks of age (rather than at birth) and first assessed responses 10 weeks later (potentially missing a transient early effect, such as we observed). There were also differences in the assays used: Jones *et al.* used a 6-day whole blood assay, whereas we used a 24-h mononuclear cell stimulation assay.

There are currently no known correlates of protection against TB. The results from this study therefore have to be interpreted with caution. However, if our pilot results are correct, and reflect a true difference in immune response to BCG immunization between infants of mothers with and without LTBI, there may be a need to investigate the effects of treating LTBI in women of childbearing age in endemic countries on the outcome of BCG immunization in their infants.

The sample size for this pilot study was small, and many outcomes were analysed, hence it is possible that some of our findings could be a consequence of multiple testing. Therefore, our initial results need to be explored further in studies with a larger sample size, and this must also be sufficient to allow adjustment for potential confounders for effects of LTBI. Early, as well as longer term, time points must be included. Small blood volumes were obtained from these infants and these yielded low cell numbers. Low frequencies of mycobacteria-specific T cells imply the need to acquire reasonably large numbers of cells during flow cytometry, and fresh whole blood, processed quickly, is likely to be more informative than frozen cells [28].

In conclusion, our results suggest that mycobacteria-specific antibodies in these infants decay rapidly, and that maternal infection with *M. tuberculosis* is associated with lower infant T-cell responses to BCG immunization. Confirmation of these results will guide the use of BCG and newly developed vaccines in TB endemic areas.
